# Supplementary material for: Illuminating the complete ß-cell mass of the human pancreas- signifying a new view on the islets of Langerhans
Source: Nat Commun. 2024 Apr 18;15:3318. doi: 10.1038/s41467-024-47686-7 (PMC11024155; doi:10.1038/s41467-024-47686-7)
Supplement: Supplementary file 1 — Supplementary Information [file 41467_2024_47686_MOESM1_ESM.pdf]

Supplementary Materials for

Illuminating the complete ß-cell mass of the human pancreas - signifying a new view on the islet of Langerhans

Joakim Lehrstrand *et al.*

Corresponding author: Ulf Ahlgren, Ulf.Ahlgren@umu.se

The PDF file includes:

Figs. S1 to S16
Table S1 and S2

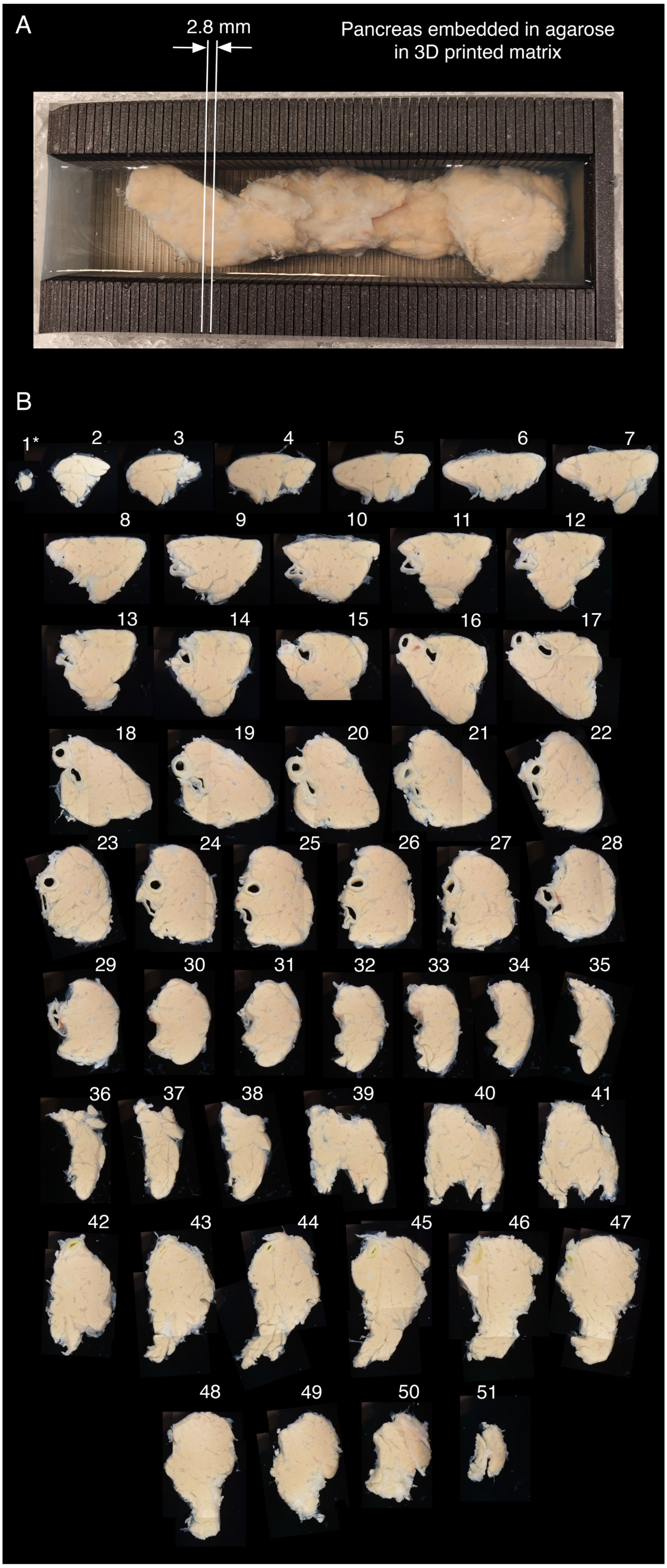

Fig. S1. Slicing and documentation of pancreatic discs for labeling and 3D analyzes. (**A**) Human pancreas embedded in an agarose support in a 3D-printed matrix (grid size 2.8 mm) The opening in the matrix (left side) makes it possible to easily remove and collect each disc. (**B**) Photomicrographs of individual discs (1-51), from tail to head regions obtained from human pancreas (e.g., H2457). *Denotes a small disc containing only 547 islets (see outlier in **Fig. 2**).

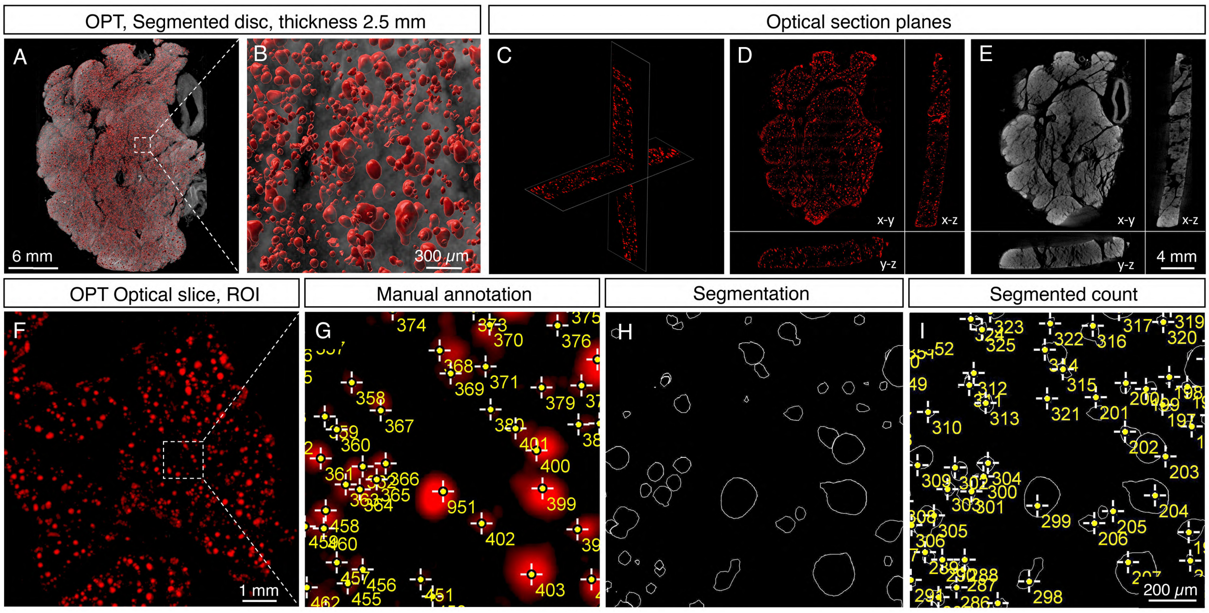

Fig. S2. Validation of antibody penetration and segmentation. (**A, B**) Segmented INS⁺ signal from OPT scan. (**C-E**) Optical section planes of the sample seen in panel **A** showing INS⁺ signal throughout the volume of the tissue (**C-D**) and of the autofluorescence (AF) signal (**E**). (**E-H**) An optical slice showing exemplifying validation of segmentation, with the INS⁺ signal observed in an optical slice ROI. Manual annotation is exemplified in panel **G**, which corresponds to the dotted box in panel **F.** The result of the segmentation pipeline is shown in panel **H** and its annotation in panel **I**. A segmentation accuracy of 7069 INS⁺ manually annotated islets resulted in a relative percentage difference of 102% (see **Table S2**).

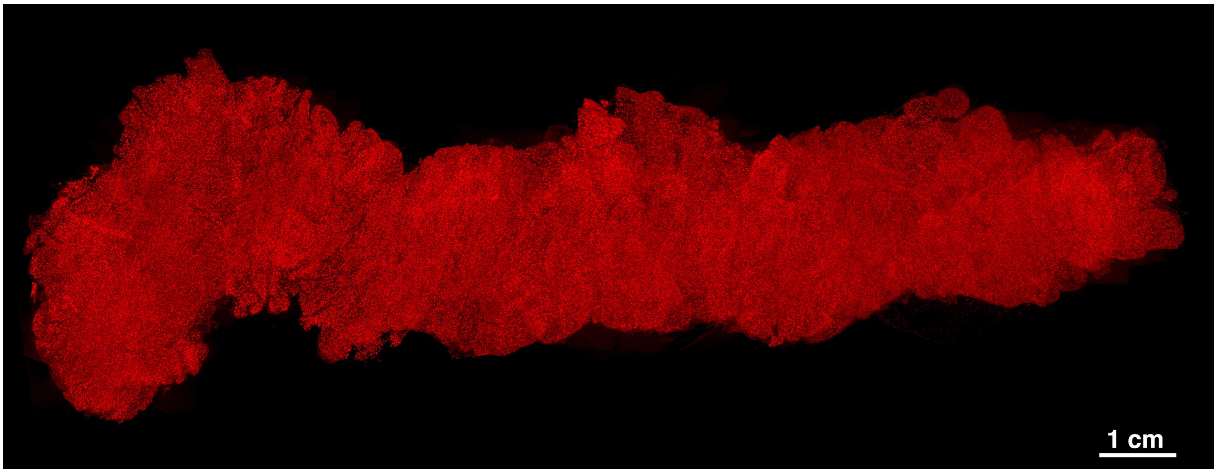

Fig. S3. Combined NIR-OPT datasets displaying the complete β-cell mass distribution of a representative human pancreas. The displayed pancreas (H2457) constitutes 45.2 cm^3 of tissue and contains 1.17 cm^3 INS^+ cells comprising 2.21 x 10^6 INS^+ islets. See also **Movie S2**. Scale bar is 1 cm.

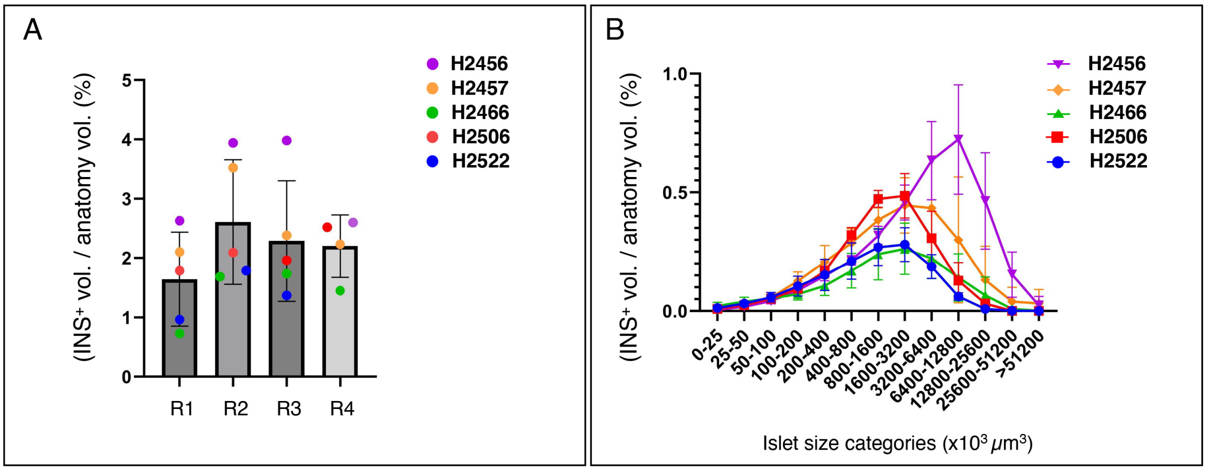

Fig. S4. 3D quantification and statistical assessment of β-cell distribution in one disc from each region (R1-4) from all donors. (**A**) Bar graph showing the INS^+ volume normalized to the tissue volume for one disc from each region (n=5 donor pancreata, n= 20 tissue discs) (**B**) Graph showing the volume of each INS^+ islet size category per donor (based on one one disc from each region) represented as volumetric density normalized to the tissue volume. Note, GraphPad robust regression and outlier removal (ROUT) with Q=1% identified H2522, R4, as an outlier and it was removed from the analysis. Note, the resolution limit of the scanner at the implemented zoom is 21μm. Error bars show mean ± SDE.

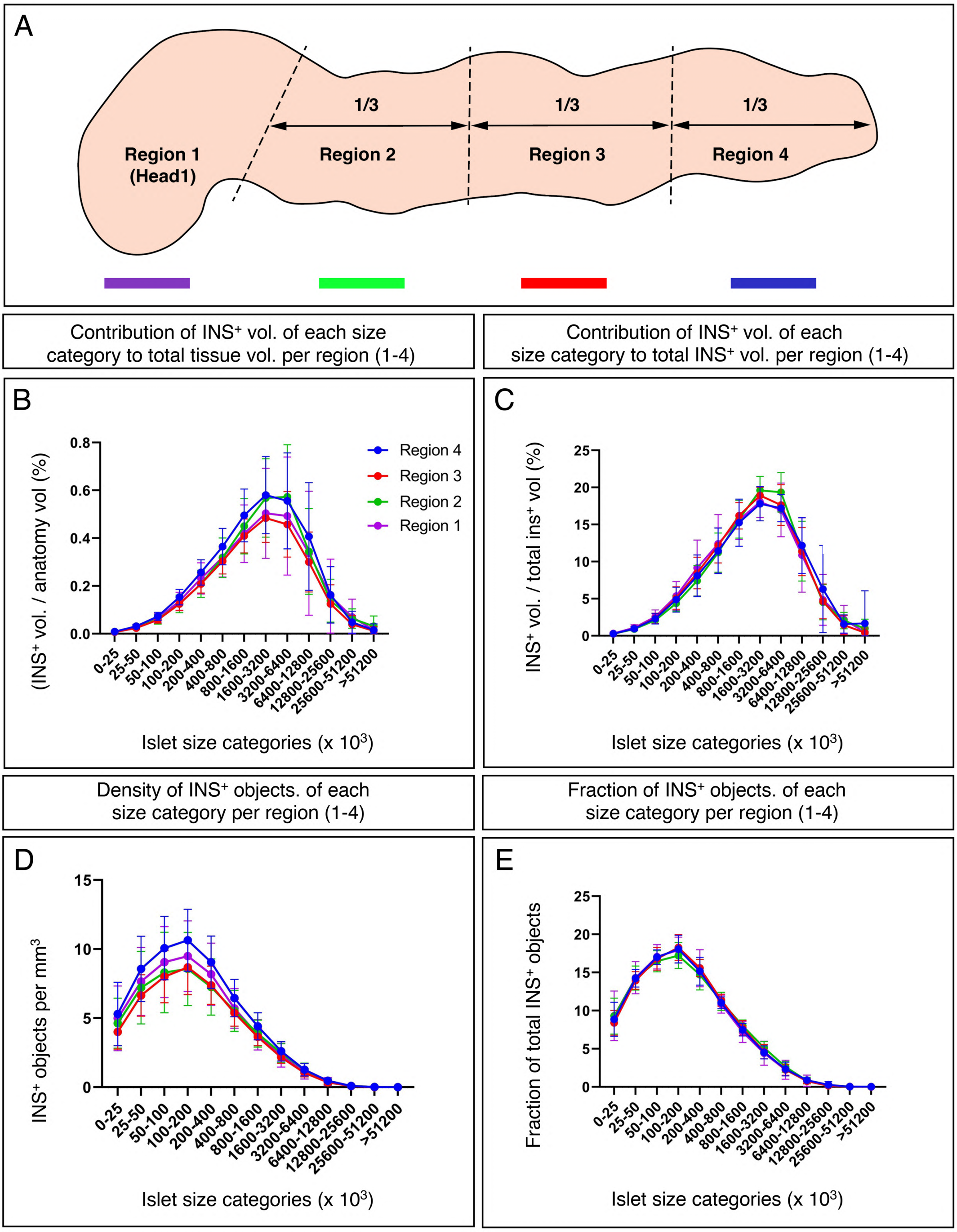

Fig. S5. Statistical analyzes of the size distribution of INS$^+$ objects per pancreatic region (1-4). (**A**) Schematic illustration of regional division of the pancreas (H2457, see main text for details). (**C-E**) For each region, graphs showing the volume of each INS$^+$ islet size category represented as (i) normalized to the entire tissue volume of each disc (n=1 donor pancreas, n= 51 tissue discs) (**B**), (ii) normalized to the entire INS$^+$ volume (**C**), (iii) the density per mm^3 per INS$^+$ islet size category (**D**) and (iv) the fraction of INS$^+$ objects per INS$^+$ islet size category (**E**). Note, the resolution limit of the scanner at the implemented zoom is 21µm. Error bars show mean ± SDE.

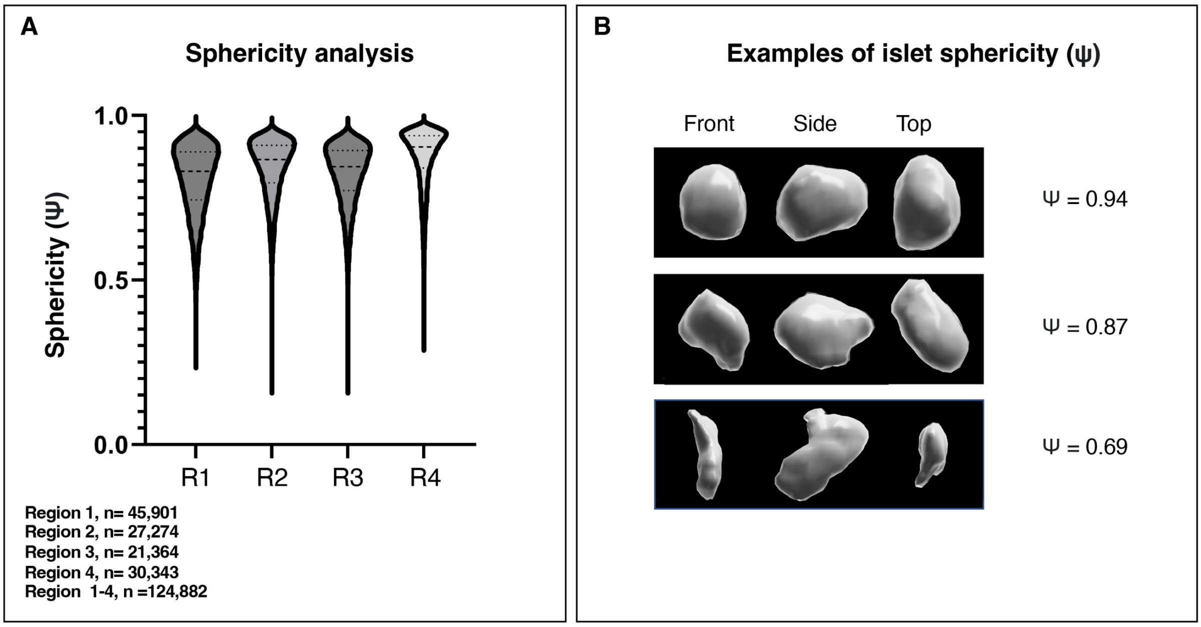

Fig. S6. Analysis of islet sphericity. (A) Violin plots showing islet sphericity and the number of islets covered by the analysis for each region (1-4), where average sphericity (Ψ) was calculated to have a value of 0.83 for a total of 124,882 INS$^+$ islets derived from H2457. (**B**) Examples of segmented islet volumes and their corresponding sphericity values (n=1 donor pancreas, n= 4 tissue discs).

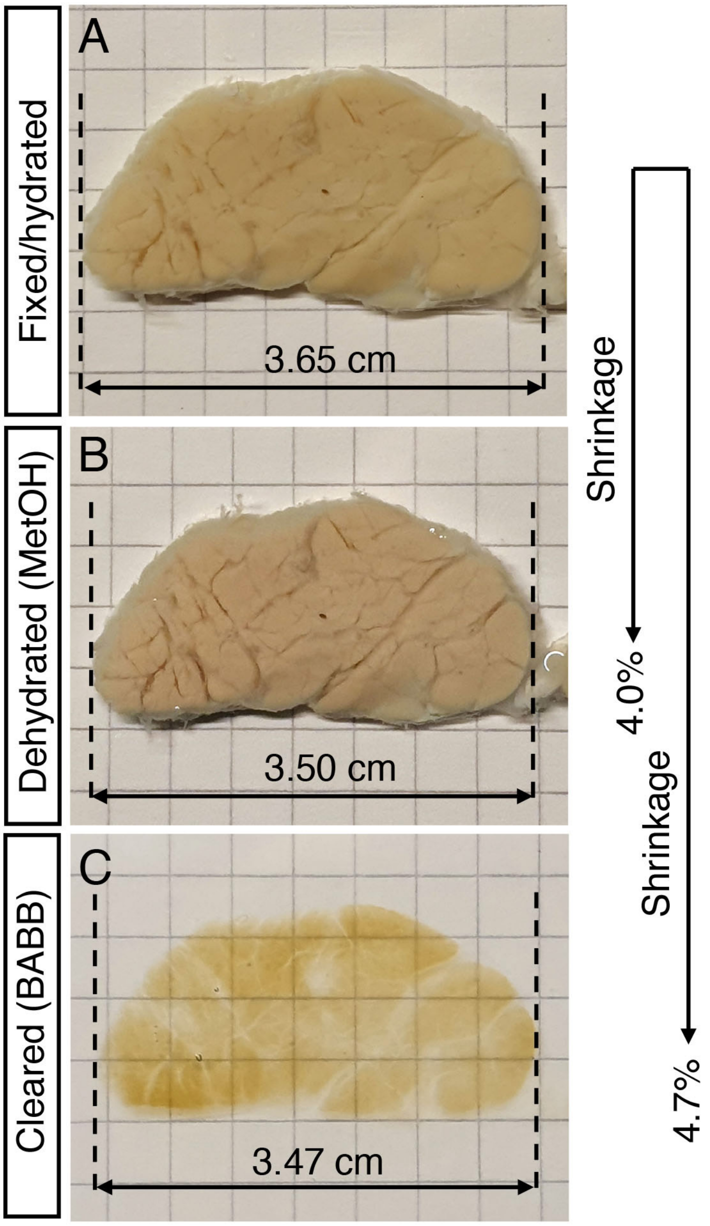

Fig. S7. Analysis of tissue processing induced tissue shrinkage of human pancreatic tissue. (A-C) Representative tissue disc illustrating the shrinkage effect of the implemented tissue processing protocols, showing the fixed disc in hydrated stage (**A**), dehydrated in MetOH (**B**) and after BABB clearing (**C**). Dehydration and clearing resulted in a 4.7% shrinkage of the tissue.

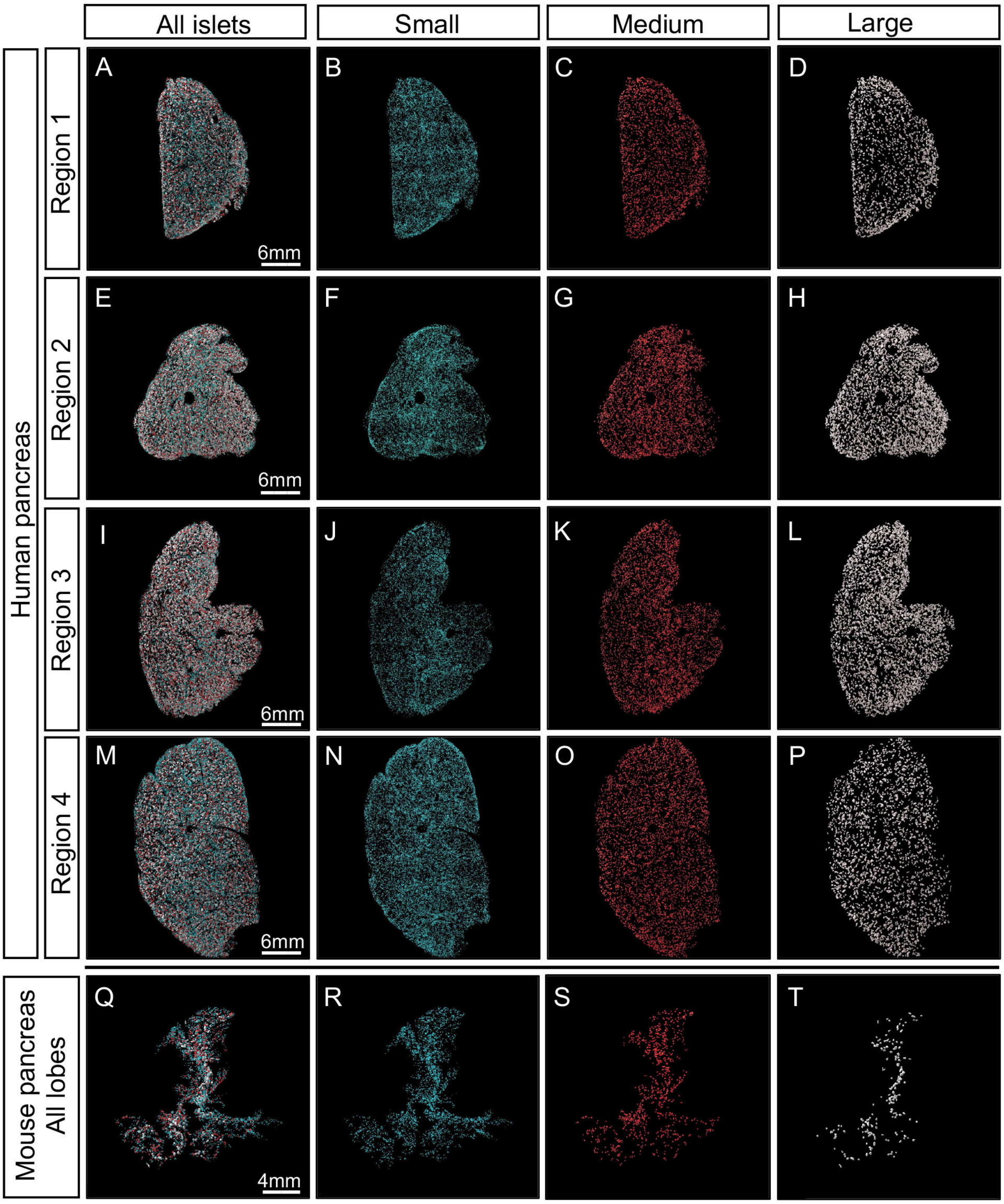

Fig. S8. Comparative distribution of islets of different size categories in human and mouse pancreas. (**A-P**) Human pancreatic discs (H2456) from region 1-4 in which the insulin signal has been segmented and pseudo colored according to size as defined in **Fig. 3**. (**Q-T**) Representative mouse pancreas from a C57BL/6 mouse at 10 weeks segmented and pseudo colored in the same way. Human islets show a more homogenous size distribution within the organ compared to mice in which large islets are primarily located along the central axis.

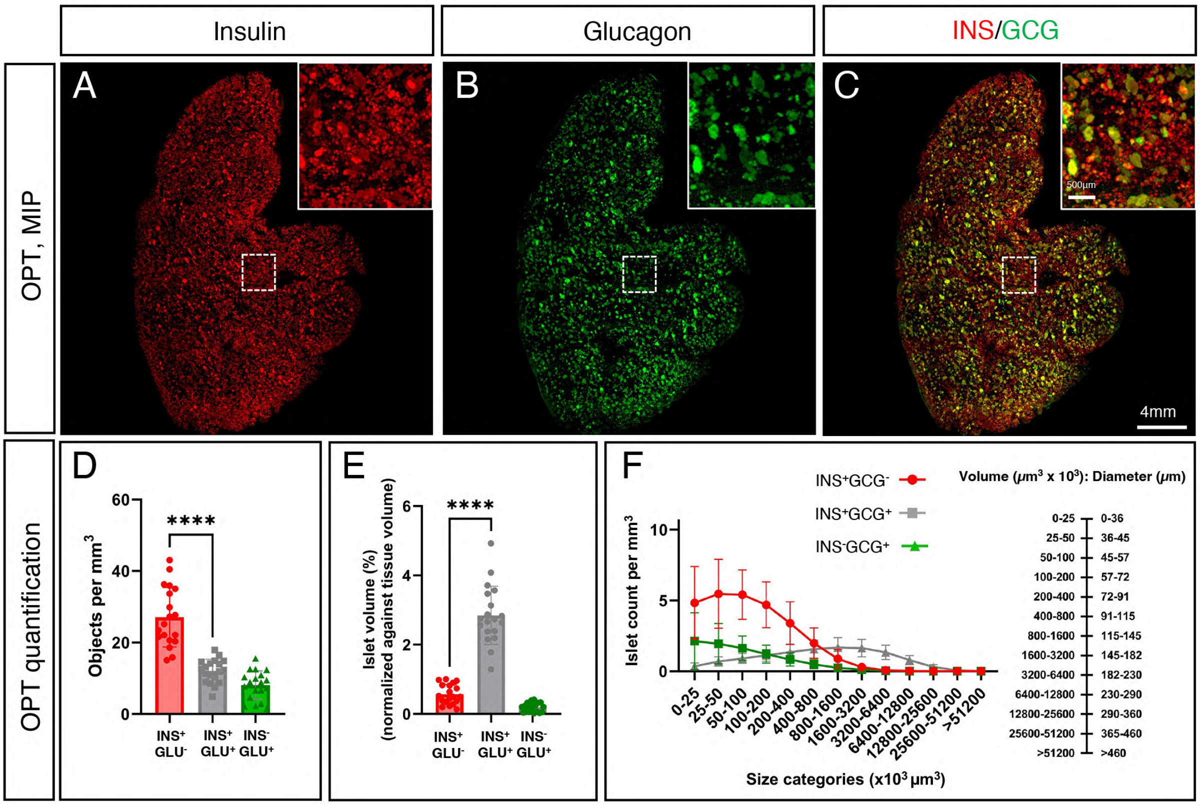

Fig. S9. OPT analyzes show that the majority of INS$^+$ islets are GCG$^-$. (**A-C**) OPT maximum projection intensity (MIP) views of representative tissue discs from an ND donor (H2456, see **Table S1**), showing insulin (**A**, red), glucagon (**B**, green) and both channels together (**C**). Note the substantial fraction of INS$^+$GCG$^-$ islets (see insets corresponding to dotted boxes in panels **A-C**). (**D**) A graph showing the average densities of INS$^+$GCG$^-$, INS$^-$GCG$^+$ and INS$^+$GCG$^+$ islets from regions 1-4 in five donor pancreata (n=5 donor pancreata, H2456, H2457, H2466, H2506 and H2522, n=20 tissue discs, **Table S1,** see also **Fig. S6**) encompassing a total of 824,154 islets. (**E**) A graph showing the volume constituted by each of the three categories displayed in **D** normalized to the total tissue volume. (**F**) Densities of all three islet subtypes (i.e., INS$^+$GCG$^-$, INS$^-$GCG$^+$ and INS$^+$GCG$^+$) in **E** represented as a function of islet size. Note that the majority of INS$^+$GCG$^-$ islets belong to the smaller size categories, with a volume typically < 200-400 x 10^3 μm^3 (=72-91 µm in diameter). Note, the resolution limit of the scanner at the implemented zoom is 21µm. GraphPad robust regression and outlier removal (ROUT) with Q=1% identified H2522, R4, as an outlier and it was removed from the analysis. Error bars show mean ± SDE. Scale bar in **C** is 4 mm (for main images in **A-C**) and scale bar in inset in **C** is 500 µm for insets in **A-C**. **D-E,** two-tailed paired t-test **** (p<0,0001).

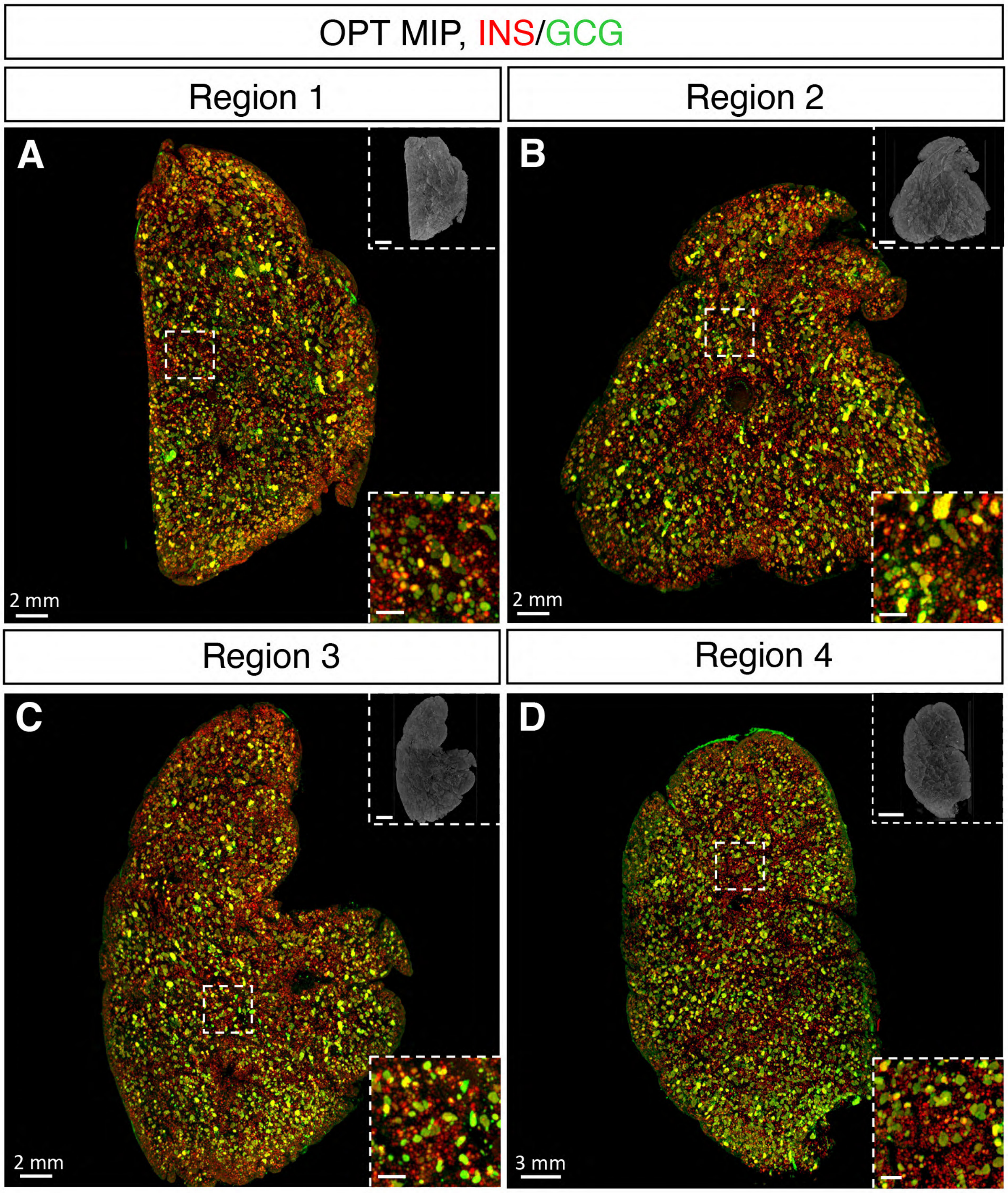

Fig. S10. OPT images of INS and GCG in human pancreatic discs from regions 1-4. (**A-D**) Representative OPT maximum intensity projection (MIP) images from scans from regions 1-4 (H2456), showing insulin (INS, red) and glucagon (GCG, green). Top insets show disc anatomy based on tissue autofluorescence (grey) whereas bottom insets, corresponding to the dotted boxes, highlight smaller islets that merely express insulin. Scale bars in top insets are 4mm in (**A-C**) and 6mm in (**D**). Scale bars in bottom insets are 500 µm in (**A-D**).

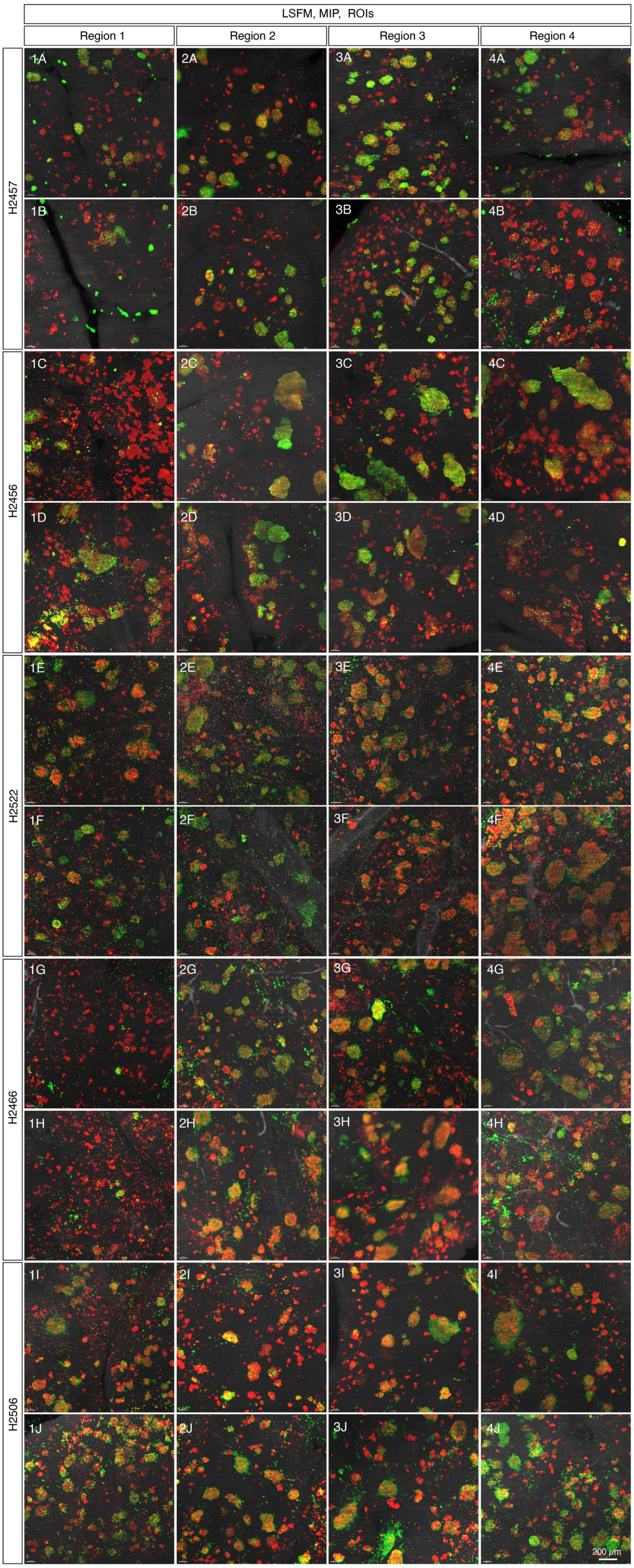

Fig. S11. LSFM analyzes of insulin (INS) and glucagon (GCG). LSFM analyzes of ROIs (sampled from H2456, H2457, H2466, H2506 and H2522, see **Table S1**) used for statistical assessment of islet heterogeneity (see **Fig. 4**) showing insulin (INS, red) and glucagon (GCG, green). Scan depth was 1 mm. Scale bar in bottom right panel is 200 μm in all panels.

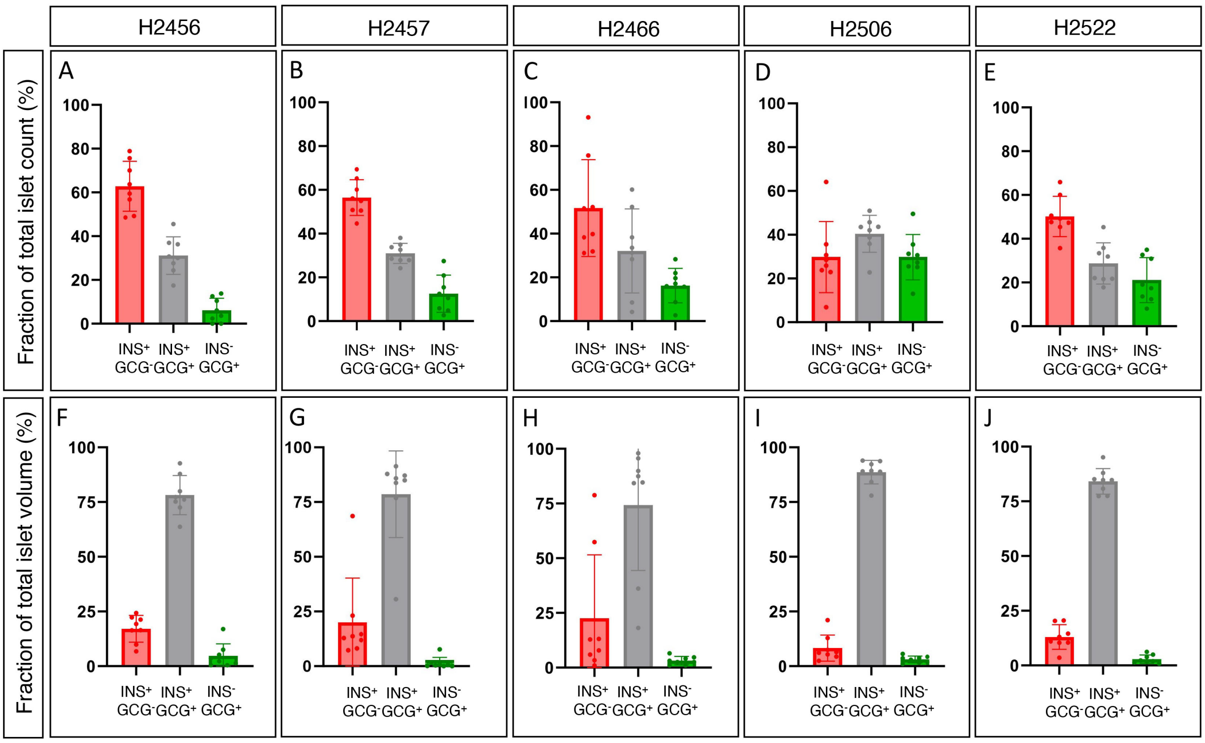

Fig. S12. LSFM quantification of INS and GCG islet heterogeneity. Graphs showing (**A-E**) the average fraction contribution of islets with < 1% GCG+ volume to the total number of INS+GCG-, INS-GCG+ and INS+GCG+ islets and (F-J) the contribution of islets with < 1% GCG+ volume to the total volume of INS- and GCG-expressing cells divided per donor (see Fig. 4 F-G). n=5 donors, n=8 independent ROIs per donor (see Table S1 and Fig. S9) scanned at 1.9 µm X-Y spatial resolution. Note, a filter was introduced in the volume quantification to exclude islets corresponding to spherical objects with a diameter of less than 29 µm (see methods). Error bars show mean ± SDE.

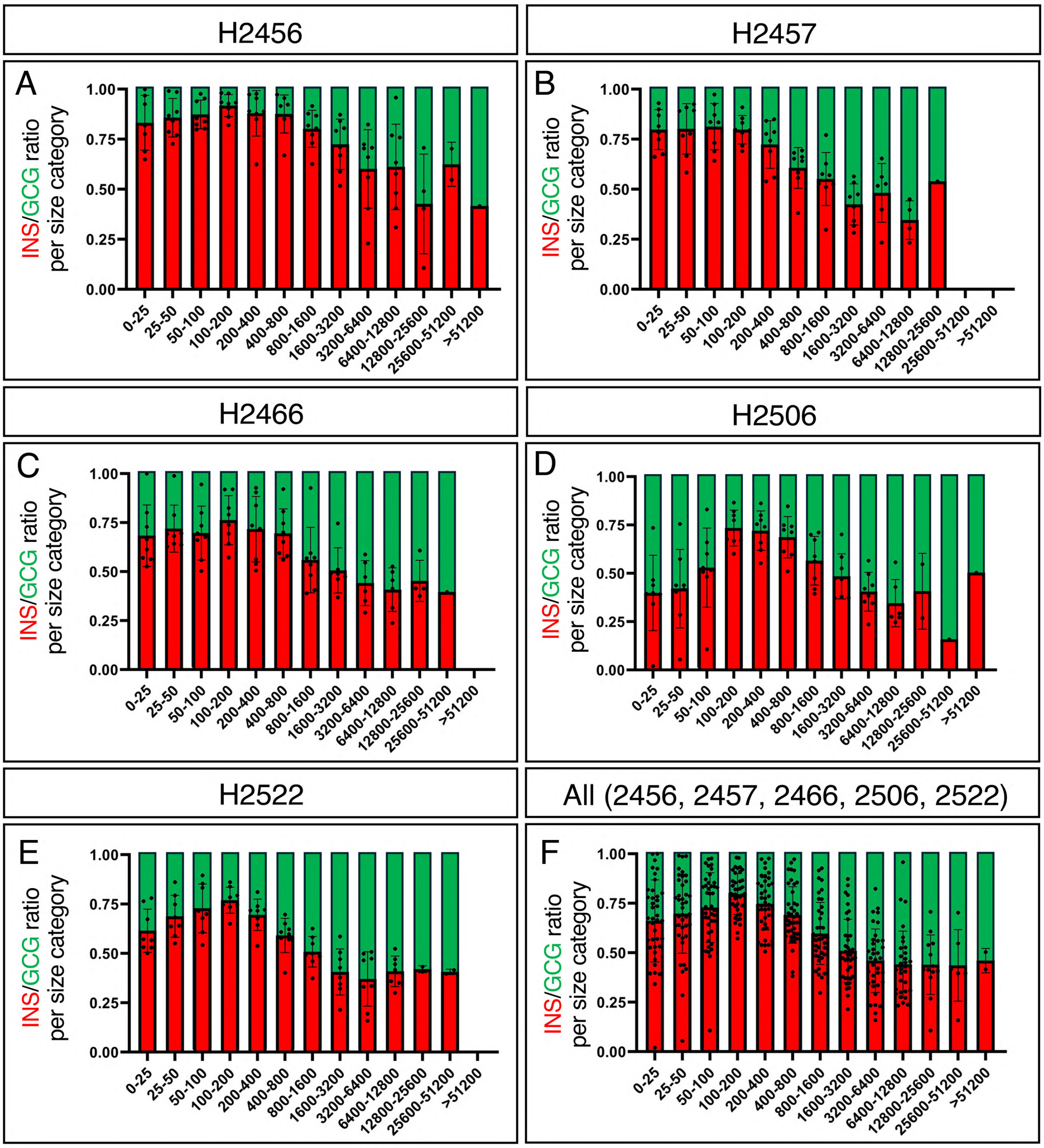

Fig. S13. LSFM quantification of INS and GCG islet composition per islet size category. (**A-F**) Graphs showing average volume ratio of INS/GCG per islet size category for each donor (**A-E**) and combined (**F**). n=5 donors, n=8 independent ROIs per donor (Table S1 and Fig. S9) scanned at 1.9 µm X-Y spatial resolution. Note, a filter was introduced in the volume quantification to exclude islets corresponding to spherical objects with a diameter of less than 29 µm (see methods). Error bars show mean ± SDE.

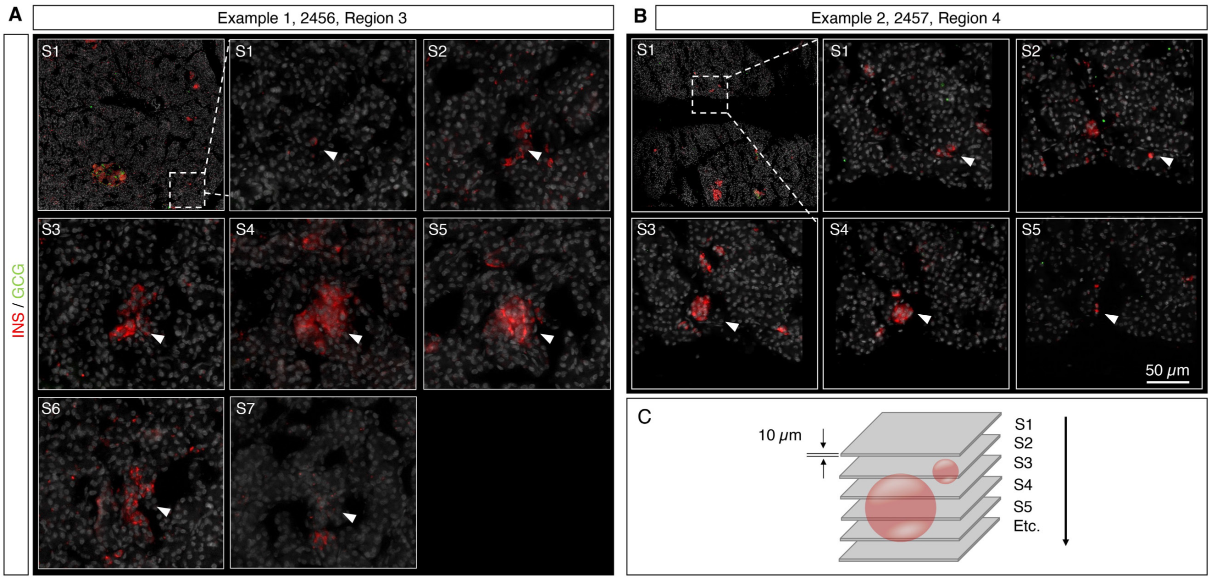

Fig. S14. Examples of Axioscan slide scanner analyzes demonstrating the absence of GCG⁺ cells in INS⁺ islets in 2D cryosections. By assessing islets as a Z-stack section (S) by section (10 µm) throughout the islet volume (white arrowhead), 3D optical data of $INS^{+}GCG^{-}$ islets were confirmed. Note the presence of GCG^{+} cells (green) in the zoomed-out view in **A** (S1, arrow) and **B** (S1, arrow). Scale bar is 50 µm.

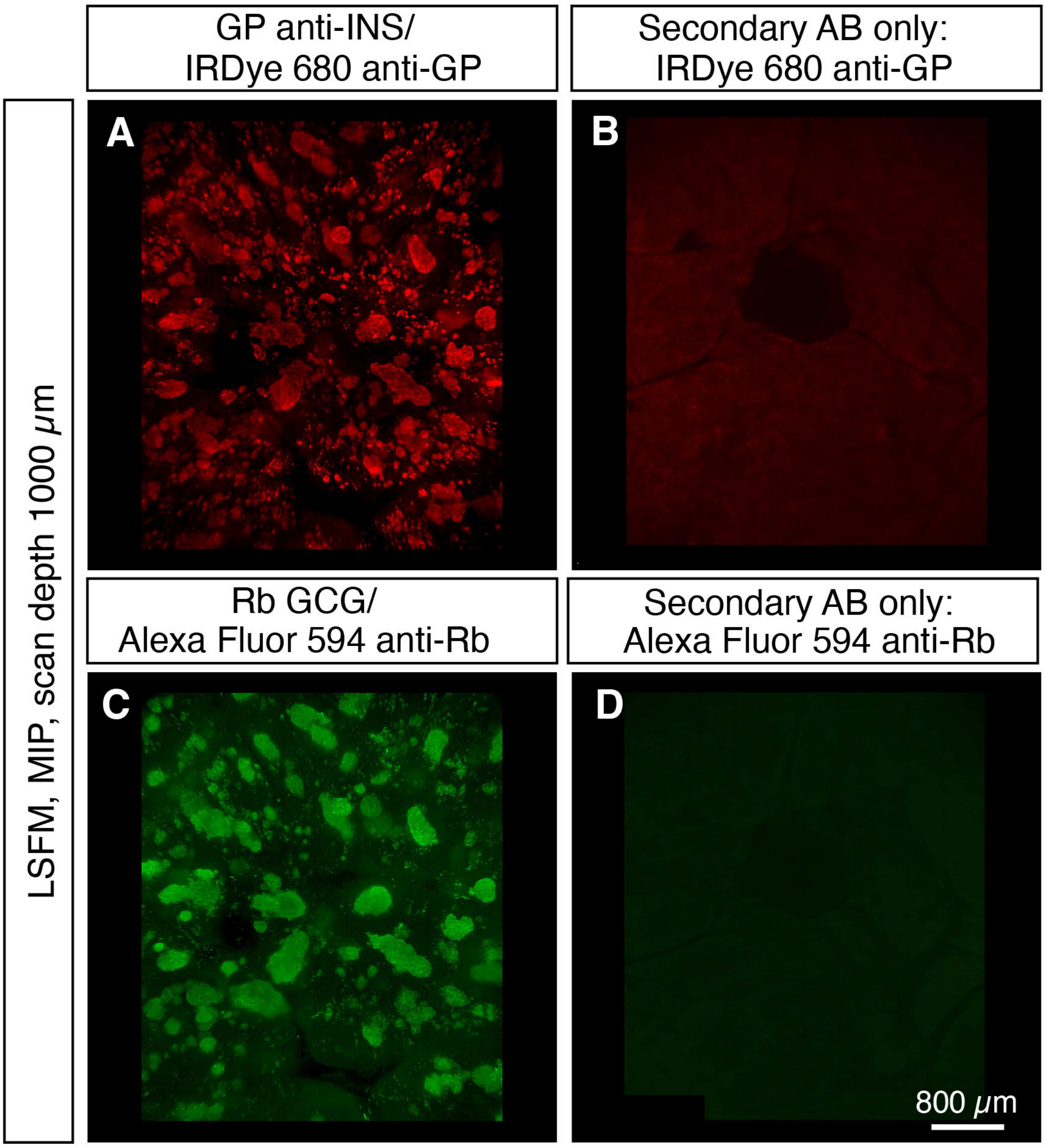

Fig. S15. Antibody controls. LSFM datasets showing primary and secondary antibodies for staining (**A**) insulin (INS) and (**C**) glucagon (GCG), as well as secondary antibodies only controls in panels (**B**) (anti-guinea-pig IgG, 680) and (**D**) (anti-rabbit IgG, 594). In all cases, antibody incubation times and scan settings were identical.

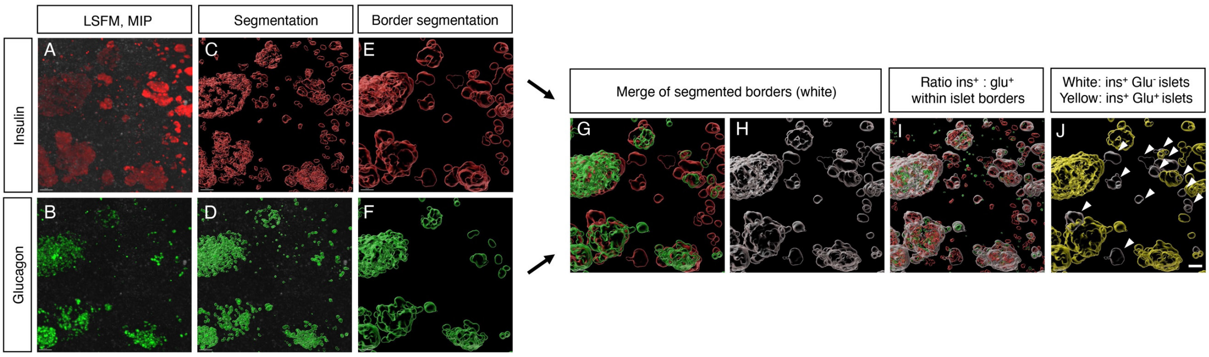

Fig. S16. Flow chart exemplifying the pipeline for LSFM-based assessment of INS:GCG ratios in human pancreatic islets. (**A**, **B**) LSFM maximum intensity projection (MIP) view of INS (**A**, red) and GCG (**B**, green). (**C, D**) Segmentation of the INS$^+$ (**C**) and GCG$^+$ (**D**) channels, respectively. (**E, F**) Segmentation of the outer border of the respective channel. (**G, H**) Segmented border showed together (**G**) and fused (**H**, white). (**I**) INS$^+$ and GCG$^+$ (red and green, respectively) within fused outer borders (white) for INS:GCG ratios. (**J)** Pseudo-coloring of segmented islet volumes >25 µm in Ø for which islets (INS$^+$ + GCG$^+$ outer border volume) containing <1% GCG$^+$ signals are pseudo-colored white, and all other islets are coloured yellow. Note that the threshold was set to 1%, primarily to exclude any inclusion of general signal/labeling noise (see methods). Scale bar in (**J**) is 50µm in (**A-J**).

Donor	Sex	Age	BMI	HbA1c	Figures	Comment	INS$^+$GLU$^-$ Islets (%)
H2456	M	25-29	31.7	29	3A-C and I-K, 4A-E, S7A-C, S8A-P, S9A-C, S10A-D, S11C,D, S14A, S15A-D, S16A-J	Discs from regions 1-4	56,47
H2457	M	25-29	23.7	35	1A-G, S1A-B, S2A-I, S3, S10B, S11A,B, S14B	Entire pancreas	62,76
H2466	F	35-39	37.6	38	S11G,H	Discs from regions 1-4	51,69
H2506	M	20-24	23.8	34	S11I,J	Discs from regions 1-4	29.80
H2522	F	45-49	25.1	NA	S11E,F	Discs from regions 1-4	50.19

Table S1. Donor data and clinical parameters.

**Specimen 1**	**Manual annotation**	**Segmentation**	**Relative difference (%)**	**Mean relative difference (%)**
ROI 1	962	1143	+19	+2.3 (SD: +/- 8.09)
ROI 2	791	778	-2	
ROI 3	727	724	0	
**Specimen 2**				
ROI 1	655	648	-1	
ROI 2	939	931	-1	
ROI 3	627	576	-8	
**Specimen 3**				
ROI 1	671	706	+5	
ROI 2	760	746	-2	
ROI 3	937	1038	+11	

Table S2. Evaluation of accuracy between manual annotation and computational segmentation.
