# Supplementary material for: Illuminating the complete ß-cell mass of the human pancreas- signifying a new view on the islets of Langerhans
Source: Nat Commun. 2024 Apr 18;15:3318. doi: 10.1038/s41467-024-47686-7 (PMC11024155; doi:10.1038/s41467-024-47686-7)
Supplement: Supplementary file 3 — Description of Additional Supplementary Files [file 41467_2024_47686_MOESM3_ESM.pdf]

Description of Additional Supplementary Files

Supplementary Movie 1. Maximum projection intensity (MIP) of a pancreatic disc (H2457) showing insulin labeled islets (red) and anatomical outline based on tissue autofluoresecence (grey).

Supplementary Movie 2. Maximum projection intensity (MIP) of combined NIR-OPT datasets displaying the complete β-cell mass distribution of a representative human pancreas. The displayed pancreas (H2457) contains 1.17 cm^3 INS^+ cells comprising 2.21 x 10^6 INS^+ islets. Note, due to size limitations the movie is significantly downsized.

Supplementary Movie 3. Movie showing a disc from a representative human donor pancreas in which the insulin signal has been segmented and pseudo colored according to size. Each size category (blue, small; red, medium; white, large) corresponds to 1/3 of the total β-cell volume of the pancreas. Note, due to size limitations the movie is significantly downsized.

Supplementary Movie 4. Movie showing a representative pancreas from a C57Bl/6 mouse at 10 weeks in which the insulin signal has been segmented and pseudo colored according to size. Each size category (blue, small; red, medium; white, large) corresponds to 1/3 of the total β-cell volume in 5 animals. In mice, large islets are predominantly distributed along the central axis following the main pancreatic duct. Note, due to size limitations the movie is significantly downsized.

Supplementary Movie 5. Maximum intensity projection (MIP) from a LSFM scan of a representative ROI (see methods) from a ND donor pancreas stained for insulin (INS, red) and glucagon (GCG, green) showing the presence of INS^+GCG^- islets.
