# Supplementary material for: Illuminating the complete ß-cell mass of the human pancreas- signifying a new view on the islets of Langerhans
Source: Nat Commun. 2024 Apr 18;15:3318. doi: 10.1038/s41467-024-47686-7 (PMC11024155; doi:10.1038/s41467-024-47686-7)
Supplement: Supplementary file 9 — Reporting Summary [file 41467_2024_47686_MOESM9_ESM.pdf]

nature portfolio

Corresponding author(s): Ulf Ahlgren
Last updated by author(s): Apr 4, 2024

Reporting Summary

Nature Portfolio wishes to improve the reproducibility of the work that we publish. This form provides structure for consistency and transparency in reporting. For further information on Nature Portfolio policies, see our Editorial Policies and the Editorial Policy Checklist.

Statistics

For all statistical analyses, confirm that the following items are present in the figure legend, table legend, main text, or Methods section.

n/a	Confirmed	
☐	☒	The exact sample size (*n*) for each experimental group/condition, given as a discrete number and unit of measurement
☐	☒	A statement on whether measurements were taken from distinct samples or whether the same sample was measured repeatedly
☐	☒	The statistical test(s) used AND whether they are one- or two-sided<br>*Only common tests should be described solely by name; describe more complex techniques in the Methods section.*
☒	☐	A description of all covariates tested
☐	☒	A description of any assumptions or corrections, such as tests of normality and adjustment for multiple comparisons
☐	☒	A full description of the statistical parameters including central tendency (e.g. means) or other basic estimates (e.g. regression coefficient) AND variation (e.g. standard deviation) or associated estimates of uncertainty (e.g. confidence intervals)
☐	☒	For null hypothesis testing, the test statistic (e.g. *F*, *t*, *r*) with confidence intervals, effect sizes, degrees of freedom and *P* value noted<br>*Give P values as exact values whenever suitable.*
☒	☐	For Bayesian analysis, information on the choice of priors and Markov chain Monte Carlo settings
☒	☐	For hierarchical and complex designs, identification of the appropriate level for tests and full reporting of outcomes
☒	☐	Estimates of effect sizes (e.g. Cohen's *d*, Pearson's *r*), indicating how they were calculated

Our web collection on statistics for biologists contains articles on many of the points above.

Software and code

Policy information about availability of computer code

Data collection	In-house developed NIR-OPT software (Eriksson et al., JoVE, 2013) LabVIEW version 20.0f1 (National Instruments, USA), ImspectorPro, version 7.1.15 (LaVision Biotec GmbH, Germany), ZEN (blue edition) microscopy software (version 3.7.97; ZEISS, Germany)
Data analysis	Scripts used for processing OPT data including alignment of axis of rotation post-OPT scanning (DFTA) (Cheddad et al., IEEE Trans. Med. Imaging, 2012) and Contrast Limited Adaptive Histogram Equalization (CLAHE) (Hörnblad et al., Islets 2011) are available as a compiled software package (together with video instructions on their implementation) at GitHub, https://github.com/ARDISDataset/DSPOPT. Other software used were; DataViewer, version 1.5.6.2 (SkyScan, Bruker microCT), NRecon, version 1.7.0.4 (SkyScan, Bruker microCT), Imaris File Converter x64, version 10.0.0 (Bitplane, UK), Imaris x64, version 10.0.0 (Bitplane, UK), ZEN (blue edition) microscopy software (version 3.7.97 ZEISS, Germany).

For manuscripts utilizing custom algorithms or software that are central to the research but not yet described in published literature, software must be made available to editors and reviewers. We strongly encourage code deposition in a community repository (e.g. GitHub). See the Nature Portfolio guidelines for submitting code & software for further information.

Data

Policy information about availability of data

All manuscripts must include a data availability statement. This statement should provide the following information, where applicable:

- Accession codes, unique identifiers, or web links for publicly available datasets
- A description of any restrictions on data availability
- For clinical datasets or third party data, please ensure that the statement adheres to our policy

Hahn, Max et al. (2022). Quantitative 3D OPT and LSFM datasets of pancreata from mice with streptozotocin-induced diabetes: Dataset 1 [Dataset]. Dryad. https://doi.org/10.5061/dryad.51c59zw8g
Raw and processed imaging datasets acquired by NIR-OPT and LSFM for all samples displayed are available from the authors upon request .

Research involving human participants, their data, or biological material

Policy information about studies with human participants or human data. See also policy information about sex, gender (identity/presentation), and sexual orientation and race, ethnicity and racism.

Reporting on sex and gender	The scope of this non-clinical study was to analyze the composition of the non-diabetic human pancreas. A balanced amount of samples from both sexes was (given the scarce availability of such samples) included in the study. Separate sex- and gender based analysis was not conducted.
Reporting on race, ethnicity, or other socially relevant groupings	N/A
Population characteristics	Donor identifier/Sex/age/BMI/HbA1c: H2456/M/25-29/31.7/29, H2457/M/25-29/23.7/35, H2466/F/35-39/37.6/38, H2506/M/20-24/23.8/34, H2422/f/45-49/25.1/NA.
Recruitment	N/A
Ethics oversight	Consent for organ donation for use in research was obtained from the donor prior to death via the Swedish National Donor Registry (https://www.socialstyrelsen.se/en/apply-and-register/join-the-swedish-nationaldonor-register/) or from relatives of the deceased donors conferred by the donor's physician and documented in their medical records. The study was approved by the Regional Ethics Committee in Uppsala, Sweden (Dnr 2017/1471-32, 2023-01845-01).

Note that full information on the approval of the study protocol must also be provided in the manuscript.

Field-specific reporting

Please select the one below that is the best fit for your research. If you are not sure, read the appropriate sections before making your selection.

☒ Life sciences ☐ Behavioural & social sciences ☐ Ecological, evolutionary & environmental sciences

For a reference copy of the document with all sections, see nature.com/documents/nr-reporting-summary-flat.pdf

Life sciences study design

All studies must disclose on these points even when the disclosure is negative.

Sample size	Sample size was limited based on the accessibility of human donor pancreata of roughly the same age. Given previous 2D analyses of (relative to our study) limited amounts of tissue, we included 5 pancreata, which we judged to be sufficient given that our analyses covers hundreds of thousands of islets, quantitatively and in 3D in non-diabetic donor pancreata.
Data exclusions	Exclusion criteria were not pre-established. To remove potential errogenous datapoints, outlier removal was applied and is described in relevant figures. For identification of outliers, GraphPpad robust regression and outlier removal (ROUT) with Q=1% was used.
Replication	All experimental procedures and findings were successfully replicated on n=5 independent donor pancreata in a total of n=51 tissue discs from the whole human pancreas and in n=16 tissue discs from additional donors (4 tissue discs per donor) using whole mount immunofluorescent staining and confirmed by 2D histological/fluorescent antibody labeling experiments on n=2 donors.
Randomization	No Randomization was performed for this study and covariates were not controlled for as the study was not aimed at determining the (non-diabetic) pancreas composition to any variables.
Blinding	No blinding was performed for this study since only non-diabetic pancreas was studied without any additional variables/groups/disease states.

Reporting for specific materials, systems and methods

We require information from authors about some types of materials, experimental systems and methods used in many studies. Here, indicate whether each material, system or method listed is relevant to your study. If you are not sure if a list item applies to your research, read the appropriate section before selecting a response.

Materials & experimental systems

n/a	Involved in the study
☐	☒ Antibodies
☒	☐ Eukaryotic cell lines
☒	☐ Palaeontology and archaeology
☒	☐ Animals and other organisms
☒	☐ Clinical data
☒	☐ Dual use research of concern
☒	☐ Plants

Methods

n/a	Involved in the study
☒	☐ ChIP-seq
☒	☐ Flow cytometry
☒	☐ MRI-based neuroimaging

Antibodies

Antibodies used	Primary antibodies: Guinea pig anti-insulin/pro-insulin (INS; Cat. No. 16049; Progen Biotechnik, Germany; diluted 1:3000) and rabbit anti-glucagon/pro-glucagon (GCG; Cat. No. HPA036761; Atlas Antibodies, Stockholm, Sweden; diluted 1:1000)<br><br>Secondary antibodies used: Donkey anti-guinea pig IRDye® 680 (Cat. No. 926-68077; Li-Cor Biosciences, USA; diluted 1:250) and donkey anti-rabbit Alexa Fluor® 594 (Cat. No. 711-585-152; Jackson ImmunoResearch, UK; diluted 1:500).
Validation	Both primary and secondary antibodies were titrated and validated on positive control sections before application. Negative secondary antibody controls were performed on pancreas control tissue. See supplementary materials.

Plants

Seed stocks	*Report on the source of all seed stocks or other plant material used. If applicable, state the seed stock centre and catalogue number. If plant specimens were collected from the field, describe the collection location, date and sampling procedures.*
Novel plant genotypes	*Describe the methods by which all novel plant genotypes were produced. This includes those generated by transgenic approaches, gene editing, chemical/radiation-based mutagenesis and hybridization. For transgenic lines, describe the transformation method, the number of independent lines analyzed and the generation upon which experiments were performed. For gene-edited lines, describe the editor used, the endogenous sequence targeted for editing, the targeting guide RNA sequence (if applicable) and how the editor was applied.*
Authentication	*Describe any authentication procedures for each seed stock used or novel genotype generated. Describe any experiments used to assess the effect of a mutation and, where applicable, how potential secondary effects (e.g. second site T-DNA insertions, mosiacism, off-target gene editing) were examined.*
